# Nanomaterials responding to microwaves: an emerging field for imaging and therapy

**DOI:** 10.1039/d0na00840k

**Published:** 2021-04-01

**Authors:** Annah J. Wilson, Mohammed Rahman, Panagiotis Kosmas, Maya Thanou

**Affiliations:** School of Cancer & Pharmaceutical Sciences, King's College London, Institute of Pharmaceutical Science Franklin Wilkins Building, 150 Stamford Street London SE1 9NH UK maya.thanou@kcl.ac.uk; Department of Engineering, King's College London UK

## Abstract

In recent years, new microwave-based imaging, sensing and hyperthermia applications have emerged in the field of diagnostics and therapy. For diagnosis, this technology involves the application of low power microwaves, utilising contrast between the relative permittivity of tissues to identify pathologies. This contrast can be further enhanced through the implementation of nanomaterials. For therapy, this technology can be applied in tissues either through hyperthermia, which can help anti-cancer drug tumour penetration or as ablation to destroy malignant tissues. Nanomaterials can absorb electromagnetic radiation and can enhance the microwave hyperthermic effect. In this review we aim to introduce this area of renewed interest and provide insights into current developments in its technologies and companion nanoparticles, as well as presenting an overview of applications for diagnosis and therapy.

## Abstract 2

In recent years, new microwave-based imaging, sensing and hyperthermia applications have emerged in the field of diagnostics and therapy.

## Graphical abstract

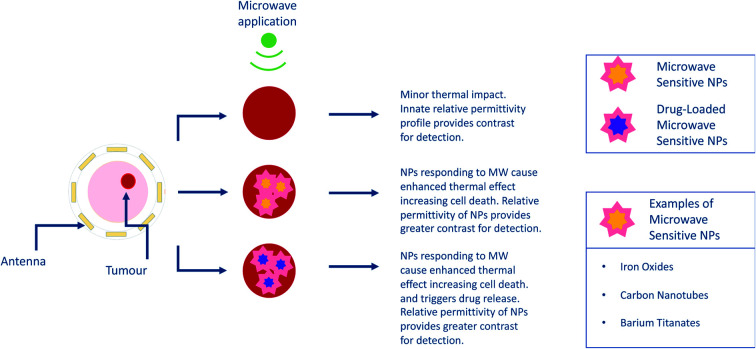

## Introduction

**Fig. 1:**
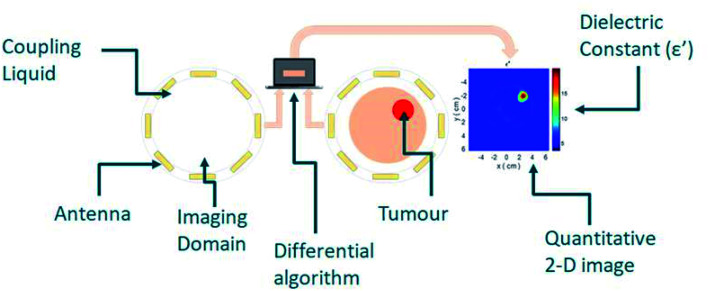
Typical microwave imaging scheme for biomedical applications. Antennas are placed around the exterior of the imaging domain which contains the object of interest, with downsteam algorithm processing allowing the identification of the target.

Nanomaterials that respond to electromagnetic (EM) radiation have been suggested for either imaging and/or hyperthermia. These nanomaterials would need to be suitably biocompatible as well as able to interact with EM energy to induce a signal response or increase the temperature locally. To this end, various nanomaterials have been suggested as contrast agents in microwave (MW) imaging and sensing, with the aim to increase contrast between the target and background tissue (Fig. 1).

This principle is based on the hypothesis that nanoparticles (NPs) are accumulated in tumours. If NPs with a high dielectric constant could accumulate in tumours, they could alter the permittivity of the tumour, providing a required tissue dielectric contrast. In recent years, however, the potential of preferential NP accumulation has been debated amongst experts. For example, a review of 232 data sets conducted by Chan in 2016 suggested that a median of 0.7% of the systemically injected dose reaches the tumours,^1^ while the same group showed in 2020 that the injected NP dose is critical. In particular, an intravenous administration of over 1 trillion NPs in mice resulted in an increased accumulation in tumours of up to 12% of the injected dose.^2^

Targeting NPs to cancer has long been considered a challenge.^3^ While an extensive review of the topic is beyond the scope of this review, it is important to consider the main factors that are important to improve the NP concentration in tumours. This is of particular significance owing to the likelihood that both imaging/sensing and hyperthermia will be dependent on the concentration of MW responsive NPs in tumours. Adaptation of the NP size to compliment tumour pathophysiology has been demonstrated to improve permeation. Another classic method to improve the concentration of nanoparticles in tumours is by modifying the nanoparticle surface to represent ligands that facilitate binding on receptors related to the lesion. A number of molecules have been shown to improve nanoparticle binding in tumours such as antibodies, peptides, sugars aptamers and small molecules.^4^

Finally, physical non-invasive methods such as localised sound and heat can increase tumour blood vessel permeability and enhance extravasation in tumours.^5^ Developing nanomaterials for MW applications should consider both the chemical and physical methods of increasing the nanoparticle concentration in tumours.

The concept of increasing the relative permittivity of tissue using nanomaterials for sensing and imaging has been introduced recently with current studies mainly using phantoms,^6–9^ and only a few published studies seen to present *in vivo* data. In hyperthermia, however, combining NPs with MW applicators has been tested *in vivo*, producing strong evidence that there is synergy. This review will present the principles of MW imaging and hyperthermia and nanomaterials that have been explored until now. The outlook of combining nanomaterials and MW modalities will be discussed.

## Microwave imaging/sensing and its principles

MW imaging or sensing can be described as the use of non-ionizing EM radiation for the profiling of tissues. Principally, antennas are used to transmit EM signals within the MW frequency (300 MHz to 300 GHz), and the subsequent scattering and reflection are processed through algorithms and used to create a dielectric tissue profile.^10^

The dielectric constant of a material can be described as the ratio of the relative permittivity of a material to that of a vacuum. However, this can be described in more depth as a function of frequency as below:1
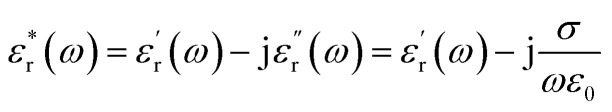
In (1), the real part 
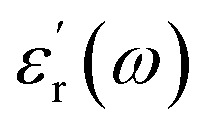
 represents how much electric energy is stored by the material, and the imaginary part 
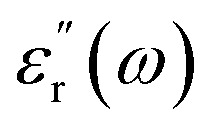
 represents the energy loss (in form of heat) due to materials properties. Another relevant quantity is the loss tangent (tan *δ*), which is also known as the loss angle, and is defined as2
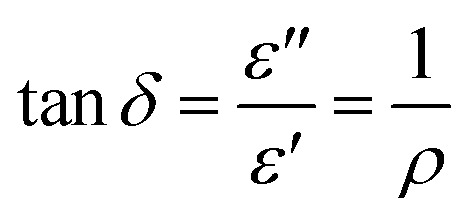


MW imaging broadly includes two approaches, termed tomography and radar. MW tomography is a quantitative imaging technique that estimates the relative permittivity of an object through cross-sectional imaging by producing a dielectric map of the target tissue.^11^ Radar-based MW imaging is a qualitative technique and focuses signals on set points to identify regions with unusual scatter.^12^ Applications of MW technologies are far reaching, with research in agricultural,^13,14^ military^15,16^ and clinical areas. This review will present the potential clinical pathways for MW imaging, which have taken both tomography and radar-based approaches and explore how nanomaterials can be used as contrast enhancers.

## Applications of microwave imaging and sensing

Microwave imaging and sensing is a complex process that is dependent on the relative permittivity of the tissue to be measured, its depth in the body and MW frequencies used to detect the difference between a lesion and healthy tissue. MW imaging faces the challenge of requirement for a high dynamic range system to successfully measure weak scattering fields.^17^ Initial studies on assessing tissues in 50 to 900 MHz frequency range indicated differences in electrical properties from normal to malignant for the kidneys about 6% and 4% and for the mammary gland about 233% and 577% average differences in permittivity and conductivity, respectively.^18^ Other types of tumours such as colon and liver showed small differences in both parameters indicating that contrast enhancers would benefit the MW detection of these tumours.

Challenges still remain open at the experimental level where there is a need to efficiently connect the microwave parameters to the target biological tissues as well as identify the suitable frequency for good resolution and penetration depth, and development of suitable contrast agents.

In MW tomography the frequency that is applicable for imaging larger objects, for example bone in whole-body imaging is 0.9 GHz whereas higher frequencies (up to 3–4 GHz) are applicable in terms of tissue penetration producing high resolution images.^19^

Breast cancer is one of the key areas in which MW technology is seeking to have clinical impact through the offering of a low-cost non-ionising imaging modality.^20^ Early work in this area identified the contrast observed in relative permittivity of healthy and cancerous tissues of the breast,^21^ providing a promising foundation for progression. Early measurements at 3.2 GHz indicated that the most common relative permittivity values for breast fat were 4 to 4.5 for normal breast glandular tissue 10 to 25, compared with 45 to 60 for breast malignant tissues.^22^ Since then, several studies have further confirmed differences between healthy and malignant tissues.^10^

**Fig. 2:**
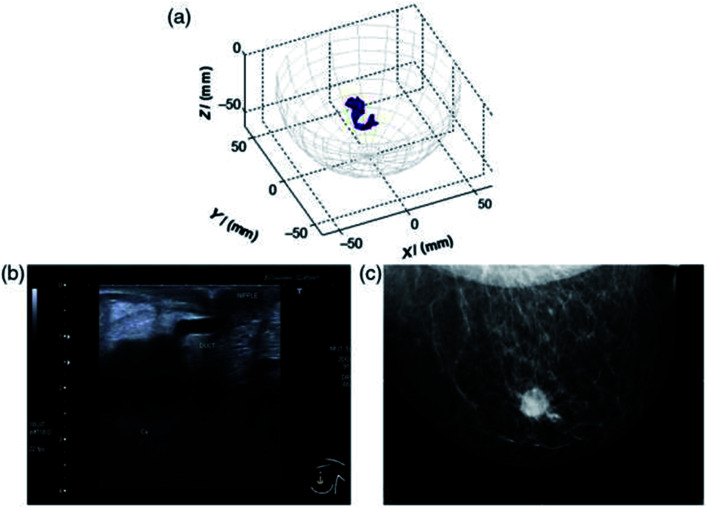
An example of a MARIA scan compared with a mammogram (MMG) and US. (a) MARIA M4 scan [max focused strength at (*X* = 3, *Y* = 21, *Z* = −42 mm)], (b) US scan, and (c) MMG. Clinical diagnoses: carcinoma 17 mm and liquid-filled milk duct. Reprinted (adapted) with permission from ref. 23 (A. W. Preece, I. Craddock, M. Shere, L. Jones, and H. L. Winton, MARIA M4: clinical evaluation of a prototype ultrawideband radar scanner for breast cancer detection, *Journal of Medical Imaging*, 20 July 2016, **3**(3), 033502, DOI: 10.1117/1.JMI.3.3.033502). Used in accordance with the Creative Commons Attribution license.

This has been strengthened by more recent clinical advances such as the MARIA® system (Micrima Ltd),^23^ which utilises a radar approach to create a 3-D image of the breast lesion (Fig. 2). The system demonstrated a detection rate of 74% across the full sample group, rising to 86% in dense breast tissue patients. For this type of breast imaging frequencies between 3 and 8 GHz are used. While there has been notable progress in this area, there are still challenges to overcome such as sensitivity and image resolution.

Feasibility studies have also assessed and found promise in the ability of MW imaging to be used on the brain,^24^ with possible applications in areas such as stroke imaging and monitoring^25,26^ as well as brain injuries. The frequency range for MW brain imaging has been reported as 0.75–2.55 GHz (ref. 27) and 0.5–4.0 GHz.^28^ As with MW breast imaging, characterisation could take either a tomographic or radar approach and aims to again provide cheap and potentially portable sensing that can ensure a very quick turn-around time, which is a critical requirement in diagnostics of acute stroke or brain injury. Clinical evaluation has been undertaken in this area, with examples including the MW helmet and accompanying diagnostic algorithm evaluated by researchers at Chalmers University in the detection of intercranial hematomas, which showed 75% specificity at 100% sensitivity.^29^ Critically, MW technology has also demonstrated the ability to differentiate between haemorrhagic and ischemic stroke, which is essential to patient treatment and outcomes.^30^ Ongoing research in this area includes prototype brain imaging scanners which are under investigation by a number of companies that develop MW imaging systems.

MW imaging has also begun to generate interest on a cellular level, with emerging studies describing the contrast between the dielectric signatures of healthy and cancerous cell lines.^31,32^ Ahmad *et al.* noted that “normal cells were observed to exhibit higher dielectric constants when compared to cancer cells from the same tissue”. In addition to this, cancer cells of different origins could be identified by their relative permittivity. It has also been suggested that MW technologies may have the potential to characterise the morphological properties of cells.^33^

## Nanoparticles and nanomaterials as potential contrast agents for microwave imaging

In recent years, NPs have demonstrated their potential to be applied extensively across biomedicine and bioimaging. In the case of MW imaging and sensing the investigation of microwave-sensitive materials looks to alter the relative permittivity properties of a targeted area, thereby providing an enhancement of the established contrast between the background and the desired area of focus. This would enhance detection through an improved reconstruction image.

A need for such contrast agents has been identified in MW imaging areas such as breast cancer. As discussed, early studies have shown the contrast observed in the relative permittivity profile between healthy and cancerous tissues of the breast. In 1988, Surowiec *et al.* profiled excised breast tumour samples from different areas with large water content variation.^21^ The results demonstrated differences in relative permittivity related to the sample origin and morphology measured at frequencies from 20 kHz to 100 MHz. While this is a promising foundation for MW imaging, due to the heterogeneous nature of the breast tissue, in some areas of the breast, such as fibroconnective and glandular tissues, this contrast can be as low as 10%,^34^ highlighting the potential for contrast agents to grow in this area. This large-scale study by the University of Wisconsin and the University of Calgary^34,35^ showed that: (i) “there is a large variation in the dielectric properties of normal breast tissues due to substantial tissue heterogeneity”^35^ and (ii) the contrast in dielectric properties “between malignant and normal adipose-dominant tissues in the breast is considerable, as large as 10 : 1”, while the contrast “between malignant and normal glandular/fibroconnective tissues in the breast is no more than approximately 10 per cent”.^34^ This highlighted the need for both improving contrast and developing of sophisticated microwave imaging approaches. With this in mind, MW imaging systems have begun to explore the inclusion of contrast enhancement, such as through iron oxides.^36^

**Fig. 3:**
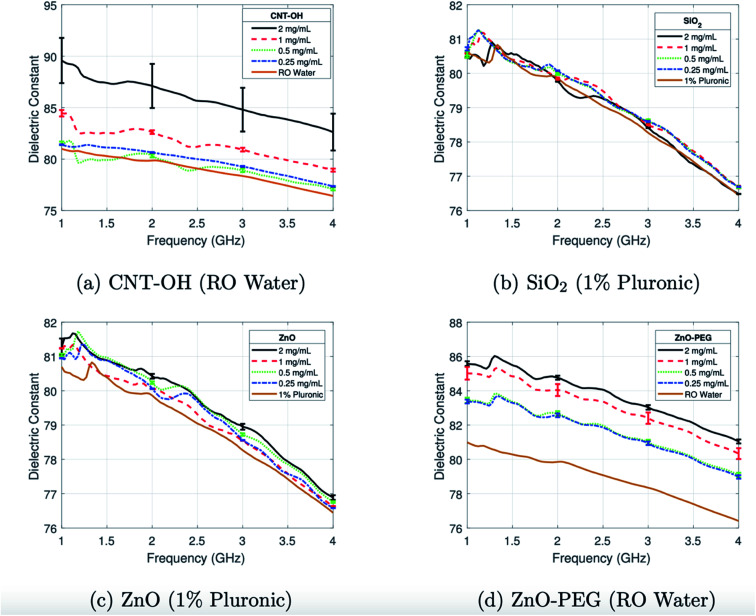
Average dielectric constant of colloidal dispersions of (a) CNT-OH in RO water, (b) SiO_2_ with 1% Pluronic, (c) ZnO with 1% Pluronic, and (d) ZnO-PEG in RO water characterised between 1 and 4 GHz. This figure has been reproduced from ref. 42 with permission from American Association of Physicists in Medicine, copyright 2018.

A range of materials have been investigated due to their capabilities to affect the relative permittivity.^37^ Iron oxides are well established in biomedicine through their use as MRI contrast agents and identification as vehicles for drug delivery.^38^ Studies have gone on to evaluate their potential use in the altering of the relative permittivity, with outcomes indicating low but detectable contrast which was hypothesised to be due to polarity either from the carboxydextran coating of the iron oxides or else the surfactant used in the preparation.^39^ Enhanced MW absorption properties have been observed in ferrite nanocomposites, such as those formed with nickel and zinc.^40^ In our group, investigations focus on ferrite NPs, with zinc ferrite particles tested in phantoms demonstrating the ability to further improve the “dielectric contrast” between healthy and target tissues when compared with commercial iron oxides.^41^ In this study, ferrites of differing zinc concentrations were synthesised and compared with iron oxides, with all particles functionalised with the polymer PMAO (poly(maleic anhydride-*alt*-1-octadecene)). These links with further work by our group conducted with ZnO particles which had been functionalized with PEG (polyethylene glycol) were demonstrated to produce a difference in the relative permittivity in a frequency range of 1 and 4 GHz when suspended in water (Fig. 3).^42^

Research in these microwave-sensitive materials has provided the foundation for contrast agent exploration, with studies moving into model systems and in some cases *in vivo* work. This has also been explored in stroke detection, where the administration of superparamagnetic iron oxide NPs was used to enhance image reconstruction through increased signal attenuation.^43^ In this pioneering study, ferumoxytol was investigated due to its potential to enhance contrast in MW imaging in phantoms in ischaemic stroke rabbit models and in humans. The study indicated that at frequencies 1–2 GHz it was possible to observe contrast in phantoms and rabbits using a purpose made MW imaging device, with increased attenuation of the MW signal in phantoms containing iron oxides. Intravenous administration of iron oxides into rabbits also produced increased MW signal attenuation above 1.3 GHz. In a healthy male human ferumoxytol provided some signal difference however the data were not clear and indicated that the signal difference was not consistent over time probably due to ferumoxytol blood kinetics. The authors concluded that considering all studies, their data suggested that superparamagnetic iron oxide NPs attenuate the transmitted MW signal and operate as MW contrast enhancers. Further benefits of this approach include the ability to functionalize NPs, allowing tissue specificity through the addition of peptides or antibodies. In 2020, Qin *et al.* explored Fe_3_O_4_ nanoparticles modified with galectin-1 antibody in the targeted microwave-induced thermoacoustic imaging of pancreatic cancer, demonstrating an enhanced contrast ratio.^44^

Carbon nanotubes (CNT) are another nanomaterial that has attracted the interest of MW researchers. An early analysis of the nanomaterial in 2009 indicated that “When carbon nanotubes are exposed to microwaves, strong absorptions are observed, producing intense heating, outgassing, and light emission”.^45^ Carbon nanotubes have unique electronic properties, which can be attributed to their distinctive structure.^46^ They have been assessed as potential MW contrast agents and have been modified in several ways to increase these properties.^47^ Composites with carbon nanotubes have also been characterised such as with the polymer polypropylene for both dielectric properties and MW heating.^48^ Although carbon nanotubes are consistent in enhancing the relative permittivity of media and phantoms, their mechanism of interacting with MW radiation at these frequencies is unclear. They do however have a notable ability to produce signal attenuation as well as consistently increase the temperature of phantom models and have therefore been suggested as agents of theranostic potential.^49^

Dielectric influencers (materials that change the dielectric properties of another material) can also be identified through their use in electronics, such as silicon dioxide (SiO_2_) nanoparticles. Often used in hybrid materials, they have several different electronic applications, such as fillers in capacitors. It comes as no surprise therefore, that SiO_2_ composites formed with a diverse range of materials including polymers and carbonyl iron, have been reported to produce an enhanced MW absorption.^50–54^ More recently, it has been indicated that SiO_2_ nanoparticles can be doped to introduce dipoles, thereby increasing their MW absorption properties.^55^

**Fig. 4:**
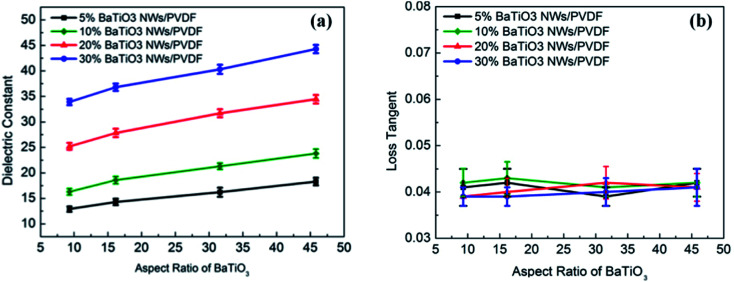
(a) Dielectric constant and (b) loss tangent of the nanocomposites measured at 1 kHz as a function of aspect ratio and volume fraction of BaTiO_3_ NWs. Reprinted (adapted) with permission from ref. 59 (H. Tang, Z. Zhou and H. A. Sodano, Relationship between the BaTiO_3_ nanowire aspect ratio and the dielectric permittivity of nanocomposites, *ACS Appl. Mater. Interfaces*, 2014 Apr 23, **6**(8), 5450–5455, DOI: 10.1021/am405038r, Epub 2014 Apr 14, PMID: 24670582). Copyright (2014) American Chemical Society.

Following a similar trend, barium titanate (BaTiO_3_) nanoparticles are also common dielectric agents in multi-layered ceramic capacitors, due to their ability to alter the dielectric properties of matrix fillers. The ability of BaTiO_3_ to increase the dielectric constant has been characterised in solution,^56^ where concentrations of BaTiO_3_ between 0 and 12% with an average size of 70 nm have shown to increase the dielectric properties of 1-propanol when in suspension at a frequency of 20.0 MHz. The dielectric constant of barium titanates has been shown to alter with temperature;^57^ at 10.0 MHz large temperature increases between 700 and 1050 °C significantly increased the dielectric constant. It has also been reported that these properties can be altered, such as through polymer modification, with interesting findings including the formation of a BaTiO_3_ composite with polyaniline powder causing a significant reduction in the dielectric constant.^58^ With formation of nanocomposites an established approach to change MW properties, combination with high dielectric constant polymers has been explored with examples including polyvinylidene fluoride (PVDF).^59^ This study demonstrated an ability to increase the dielectric constant of these BaTiO_3_ nanowire composites through the tuning of the aspect ratio, which was achieved though the alteration of the hydrothermal reaction temperature. A further study in the area indicated BaTiO_3_–PVDF composites could produce a higher dielectric constant when the BaTiO_3_ nanoparticles were modified with PEG, the basis for which was proposed to be due to enhanced polarisation (Fig. 4).^60^

Hydrogenation of BaTiO_3_ nanoparticles has also been studied as a mechanism to increase their MW absorption,^61^ with characterisation conducted in a paraffin wax dispersant over a 1–18 GHz frequency range. Overall, the hydrogenated particles showed increased MW absorption when compared with their pristine counterparts which the authors postulate is due to increased interfacial polarisation.

Titanium oxide (TiO_2_) nanoparticle studies have shown its high dielectric constant enhancing capabilities with studies involving polymer matrices and ceramic systems.^62–64^ As with other MW absorbing materials, it has been demonstrated that synthesis parameters such as temperature have an influence on the downstream dielectric constant.^65^ Further modification of the synthesis such as partial crystallization and hydrogenation can also have a positive impact on these properties.^66,67^ Titanium oxide has also shown early promise in cancer therapy as a method to induce necrosis of cancer cells when illuminated with specific electromagnetic radiation as well as additional radiation therapy.^68^

Studies have also been conducted using microbubbles, with the overall indication that the presence of the microbubbles decreased the dielectric properties thereby providing contrast.^69^ Although interesting, there were some limitations to this, as the dielectric behaviour was heavily influenced by the dispersion of the microbubbles (Table 1).

**Table 1:** Summary of data for microbubbles assessed at 3 GHz. Adapted from ref. 69. A. Mashal, J. H. Booske and S. C. Hagness, Toward contrast-enhanced microwave-induced thermoacoustic imaging of breast cancer: an experimental study of the effects of microbubbles on simple thermoacoustic targets, *Phys. Med. Biol.*, 2009, **54**(3), 641–650, DOI: 10.1088/0031-9155/54/3/011, © Institute of Physics and Engineering in Medicine. Reproduced by permission of IOP Publishing. All rights reserved

Solution	Initial microbubble concentration (% by weight) at 0 min	Estimated actual concentration (% by weight) at 2.5 min	Average *ε*_r_ (3 GHz)	Average *σ* (3 GHz) (S m^−1^)
1	0	0	14.03	2.32
2	5	20	9.66	1.33
3	10	30	8.34	1.11
4	20	35	7.38	0.95
5	30	40	6.86	0.84
6	100	100	NA	NA

Table 2 shown below shows a selection of nanomaterials identified for MW imaging and that have not been identified as contrast agents in other imaging modalities. These nanomaterials represent a variable chemical composition with a minimum change of surface characteristics. The surface of nanomaterials should be altered to allow biocompatibility and tumour receptor potential.

**Table 2:** Examples of nanomaterials tested for MW imaging and sensing that represent a variable chemical composition

Nanomaterial type	Composition	Size	Shape	Surface	References
Ferrites	Nanocomposite with nickel and zinc (Ni_0.6_Zn_0.4_Fe_2_O_4_)	Average of 53 nm	Not specified	Not specified	40
	ZnO-PEG	60–100 nm	Spherical	PEG	42
Carbon nanotubes	Single-walled carbon nanotubes	Diameter 1–2 nm	Rod	Not modified	49
Barium titanates	Pristine and hydrogenated BaTiO_3_	Diameter 20–30 nm (pristine) and 100–200 nm (hydrogenated)	Not specified	Not modified	61

## Microwave hyperthermia as a cancer treatment method

In this context, hyperthermia refers to the heating of a targeted area with an end goal in the improvement of therapy, which can be induced through a range of methodologies including microwave. The use of hyperthermia in cancer treatment is an established clinical technique, where the application of heat to around 42 °C has been demonstrated to increase tumour cell death and improve the efficacy of treatment options such as chemotherapy and radiotherapy by increasing cytotoxic effects on the desired cells.^70,71^ The treatment may be applied locally, regionally or through the whole body, with aims in producing as little damage as possible to any surrounding healthy tissues. Heat is applied to induce either reversible tissue changes including the increase of cell metabolism, blood perfusion and oxygen supply *via* hyperthermia (40–45 °C), or to irreversibly change tissue during ablation (50–110 °C). The tissue change (damage) depends on both the duration and temperature, and can change blood perfusion, or cause changes in the mechanical, electrical and thermal tissue properties.^72^ Hyperthermia has also been considered as a means to enhance immunotherapy approaches to cancer treatment.^73^

As with many cancer therapeutics, specificity remains a hurdle for hyperthermia and while minimally invasive, there may be the undesired effect of heating on surrounding tissues. As applications of microwaves have been established as a means of inducing hyperthermia, one possible solution to this is through the utilisation of microwave-sensitive materials to allow targeted enhancement of the heating effect. NPs have been demonstrated to prefer highly vascularised tumours and they could offer such specificity when combined with non-invasive MW hyperthermia techniques.

Microwave hyperthermia is usually applied for superficial or intraluminal/intracavitary tumours using antennas at 433 MHz, 915 MHz and 2450 MHz. Microwave ablation is induced using a needle applicator at frequencies 915 MHz or 2.45 GHz. Microwave ablation causes dielectric heating in a volume around the applicator.^74^ Higher frequency MW heat systems use antennas with small dimensions, showing flexibility in probe design. Several studies on ablation have been conducted at higher frequencies, ranging from 9.2 to 24.1 GHz.^75^

Although the systems of non-invasive ablation are in development, endoscopic MW devices prove to be valuable tools in inducing localised ablation when used under imaging. These endoscopic devices would largely benefit from the use of nanomaterials that could enhance the local MW absorption.^76^ Devices for endoscopic ablation are in development^76^ and could find biomedical applications of treating oesophagus lung and bowel lesions. The use of MW absorbing nanoparticles with such endoscopic devices could offer a specific and controlled ablation method.

Thermometry is required in all thermal treatments. Non-invasive thermometry should be used during the treatment to provide temperature feedback in order to safely control treatment and avoid exceeding the temperature limits. From all imaging techniques used such as X-ray computed tomography, microwave tomography, echo sonography, and magnetic resonance (MR) imaging, the proton resonance frequency method of MRI is the only method currently in clinical practice due to its temperature sensitivity that appears consistent in most aqueous tissues and because it can be easily observed using common clinical MRI scanners.^77^ Proton resonance frequency shift (PRFS) is used in MRI guided interventions. The PRFS of hydrogen in water molecules, the most abundant molecules in the human body, depends linearly on temperature. PRFS can be affected by body motion, however this can be corrected. There is however indication that some paramagnetic agents can affect the PRFS and temperature measurements. Gadolinium based contrast agents (linear) have been found to affect the focal heating point read out by 2 °C. Such an effect can be corrected by the dose and or the type of contrast agents.^78^ If, however paramagnetic contrast agents affect the temperature feedback, emphasis should be given such that the nanomaterials used to increase the temperature locally after MW application do not have a large effect on PRFS.

## Nanoparticle enhanced microwave hyperthermia

MW sensitive nanomaterials have the potential to be applied in the growing area of hyperthermia with magnetic NPs already identified as being capable of hyperthermia enhancement.^79^ In this approach, the focus shifts from detection and diagnostics to harnessing the capabilities for therapeutics, both as a stand-alone technique and in combination with drug administration. The theory behind this approach involves the selective uptake of MW sensitive NPs by a target area such as a tumour. Due to the presence of these NPs, the application of MW radiation will provide enhanced hyperthermia in the target region. This has the potential to be combined with drug-loaded NPs to deliver a dual-approach therapeutic, in keeping with the current trend towards research in theranostics.^80^

**Fig. 5:**
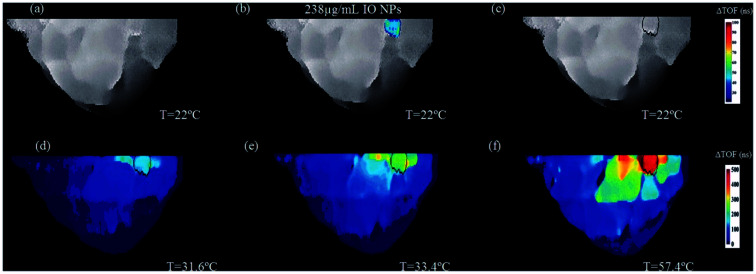
An example of two-step image guided therapeutic procedure in a heterogeneous breast phantom, using iron oxide-based NETUS (nanoparticle-enhanced transmission ultrasound). Reproduced from ref. 81 with permission from the publisher-Taylor & Francis Ltd, http://www.tandfonline.com.

As in MW sensing, breast cancer forms a key area of interest within MW hyperthermia, with studies conducted *in vitro*, *ex vivo* and using breast imaging model systems.^81^ This novel study utilised transmission ultrasound combined with NPs to monitor subsequent application of MW hyperthermia (Fig. 5). The results demonstrated the favourable impact of 10 nm Fe_3_O_4_ iron oxide nanoparticles on the induction of heat alongside improvement of target detection and non-invasive thermo-monitoring. Copper oxide particles were also evaluated for the same purpose but were not observed to improve the heating rate. Studies are not limited to breast cancer, with maghemite γ-Fe_2_O_3_ evaluated for hyperthermia potential in liver cancer. Here it was noted that the presence of the magnetic NPs allowed a lower power input of MW radiation to achieve the desired level of hyperthermia.^82^ Iron oxide composite NPs have also been identified as candidates for microwave-controlled drug release,^83^ further demonstrating their potential in both contrast and therapeutics.

**Fig. 6:**
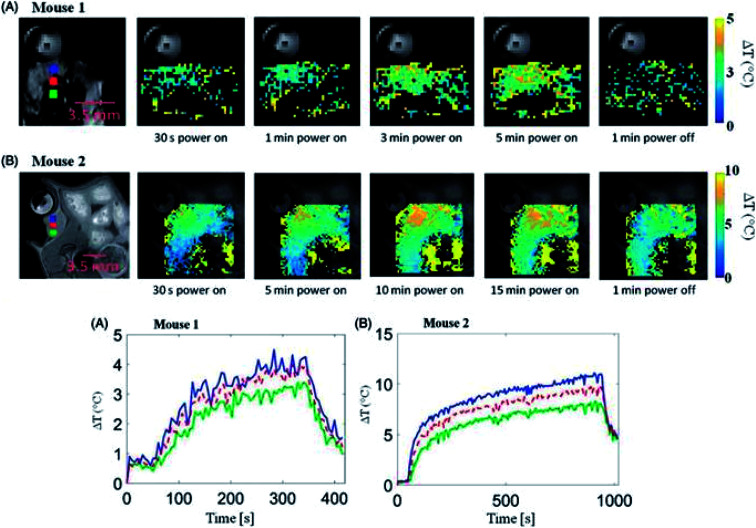
(Upper panel) Transient evolution of spatial temperature profiles during and microwave exposure *in vivo* with 20 W input power. (Lower panel) *In vivo* transient temperature profiles within subcutaneous tumours during microwave exposure. Reproduced from ref. 85 with permission from the publisher-Taylor & Francis Ltd, http://www.tandfonline.com.

In 2017, McWilliams *et al.*^84^ also explored iron oxide NPs in relation to MW hyperthermia, testing a range of compositions and spherical, cubic and hexagonal shapes. Experiments were conducted at frequencies of 2.0, 2.45 and 2.6 GHz in tissue-mimicking phantoms. Overall, the authors concluded that the spherical Fe/Fe_3_O_4_ nanoparticles showed the greatest potential for thermal enhancement. The Prakash group has also conducted *in vivo* research demonstrating MW hyperthermia systems combined with magnetic resonance thermometry, again establishing feasibility of such approaches (Fig. 6).^85^ However there are noted limitations in the MW applicators, with further work looking to model the design of more focussed applicators.^86^

Multiwalled carbon nanotubes (MWNTs) have been extensively characterised for their MW absorption properties.^87–90^ With this in mind, recent work^91^ has sought to combine these desirable properties with composite nanomaterials (comprising lithium, zinc, cobalt and iron oxide) through integration into the matrix. Further work from this group has explored the microwave properties of Ni–Zn–Co-ferrite nanoparticles that had been similarly encapsulated in MWNTs.^92^

Gold has also been investigated as a material with potential in microwave absorption and hyperthermia. Polymer nanocomposites demonstrated a higher microwave absorption when doped with gold NPs.^93^ An SU-8 polymer material was mixed with different concentrations of gold NPs and assessed over a frequency range of 0.5 GHz to 20 GHz. Of the two concentrations tested, 2.2 wt% of gold was demonstrated to be superior in the increasing of microwave absorption, with a small particle size of approximately 4 nm. Furthermore, increasing the NP concentration resulted in a more pronounced effect. This has also been observed *in vitro*, where incubation with gold NPs was observed to decrease the survival rate in cells exposed to a combination of microwave hyperthermia and chemotherapy,^94^ further inferring their microwave absorption properties. Other recent studies include gold nanoclusters, incubation with which reduced cell viability in combination with microwave radiation applied at 1 GHz.^95^ In an interesting recent study, gold nanostructures were also synthesised with solutions of honey, and subsequently evaluated for their microwave-thermal properties.^96^

Further research has also demonstrated the strength of a combination effect between microwave hyperthermia and targeted drug release, with examples including a cell model study using fullerene. In this study, cell viability was measured as an outcome of the application of fullerene which had been encapsulated by specifically designed Pluronic F127-chitosan nanoparticles. The cells were demonstrated to take up the NPs when microwave heating was applied. The overall outcomes demonstrated a more powerful effect on cell viability when the microwave heating and NPs were combined.^97^ This may be attributed to the accelerated heating time observed in the presence of fullerene and the potential for a higher intracellular temperature, which is an established effect of this agent.^98^*In vivo* studies have also been conducted with a similar theme, for example the evaluation of self-assembled micelles capable of encapsulating both chemotherapy drugs and magnetic NPs.^99^ Here, the micelles were allowed to selectively accumulate in the tumour before microwave application, with the resultant effect being the triggered drug release alongside tumour temperature increase. This appeared to have a positive impact on the tumour volume in the ensuing 19 day observation period. Such *in vivo* studies are not limited to cancer therapy, with work undertaken to evaluate targeted drug delivery for rheumatoid arthritis.^100^ This study explored the delivery of novel thermosensitive liposomes loaded with sinomenine hydrochloride paired with MW hyperthermia and demonstrated reduced arthritic scores and pathological features in the combined approach group.

Another novel approach recently described is microwave dynamic therapy. This harnesses the principles of photodynamic therapy, whereby the production of reactive oxygen species is used to destroy cells; an effect that has been noted to be more pronounced in tumour cells.^101^ Limitations include poor light penetration depths, which Wu *et al.*^102^ have sought to overcome through a combination of microwave irradiation and NPs tested both *in vivo* and *in vitro*. The NPs are zirconium dioxide composite with ionic liquid and liquid metal components and are functionalised with PEG for biocompatibility. The application of these particles demonstrated increased microwave sensitivity postulated to be due to the ionic liquid component, and this in turn meant that an increased thermal effect was seen upon microwave irradiation. Their presence also triggered free radical production which was observed to decrease cell viability. Studies such as these provide an interesting foundation for further research and highlight the capability of microwave sensitive materials in therapeutics. The potential combination with CT imaging for real-time monitoring gives an idea of how therapeutics and imaging in this area can be unified to deliver a targeted solution.

Interestingly, zirconium dioxides have also been suggested as drug delivery vehicles.^103^ In this study, ZrO_2_ nanospheres were loaded with the cancer therapeutic arsenic trioxide (which functions through the damage of the mitochondria), as well as the mitochondrial targeting ligand TPP (triphenylphosphine). The composites were also modified with tetradecanol for temperature sensitive release upon the application of microwave hyperthermia.

There are a small number of studies that suggested metal organic framework NPs for MW hyperthermia for instance, the Zr metal organic framework or the equivalent nanocube using Mn doped Zr metal organic frameworks.^104,105^ These studies clearly indicate that MW absorption by NPs can be tuned. NP chemists can design materials with high dielectric constants and conductivity that are biocompatible with tuned MW absorption properties.

As mentioned earlier, MW hyperthermia has been identified as an immunotherapy candidate. This has recently been investigated in combination with microwave-sensitive composite NPs and bovine lactoferricin; a cationic anti-cancer peptide,^106^ which has been evidenced to cause tumour cell death as well as induce an enhanced immune response. Due to degradation, application of cationic anti-cancer peptides has proved challenging. This study explores PEG-coated thermal and pH sensitive NPs to act as carriers, negating this effect. Again, the MW absorption of these materials produces an enhanced thermal effect, and we see the combined effect of the therapeutic delivery and heating in the causation of tumour cell death.

Table 3 depicts a selection of nanomaterials that have been tested with MW hyperthermia. Similar to MW imaging these materials represent a variable chemical composition.

**Table 3:** Examples of nanomaterials tested for MW hyperthermia that represent a variable chemical composition

Nanomaterial type	Composition	Size	Shape	Surface	References
Iron oxides	Magnetite Fe_3_O_4_	10 nm (in water)	Spherical	Not specified	81
	Core/shell Fe/Fe_3_O_4_	10 nm; 20 nm	Spherical	Dopamine-coated	84
Multiwalled carbon nanotubes (MWNTs)	Li_0.3_Zn_0.3_Co_0.1_Fe_2.3_O_4_ doped MWNT	Average matrix diameter 30–50 nm (range of NP crystallite sizes)	Rod	Not specified	91
	Ni–Zn–Co-ferrite doped MWNT	Average matrix diameter 30–50 nm (NP crystallite size of ∼34.7 nm)	Rod	Not specified	92
Gold	SU-8 polymer nanocomposites doped with gold NPs	Approximately 4 nm	Spherical	Not specified	93
	Gold NPs	20 nm; 40 nm	Spherical	Not specified	94
Zirconium	Zirconium dioxide composite with ionic liquid and liquid metal components	210 ± 60 nm	Spherical	PEG	102
	Zirconium metal organic framework doped with Mn	Average size of 60 nm	Nanocubes	PEG-(NH_2_)_2_	104

## Summary and outlook

**Fig. 7:**
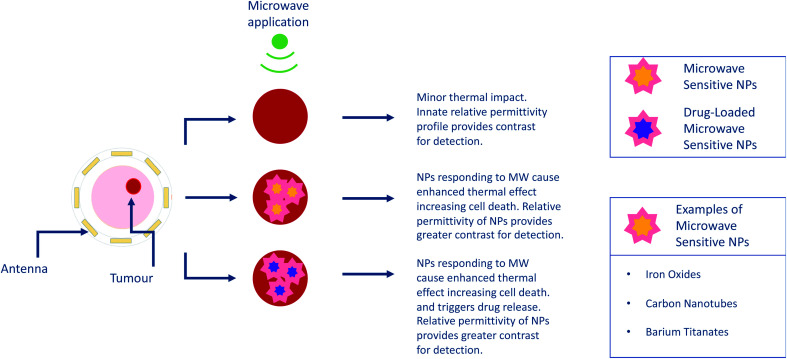
Overview of the use of nanoparticles in microwave imaging/sensing and hyperthermia.

The development of medical devices for imaging and sensing (see Fig. 7) is a significant multidisciplinary mission that suggests a holistic approach. Achieving this will require a combination of theoretical physical and biological knowledge, along with an understanding of the regulatory and market adoption avenues. In the event that these modalities adopt companion nanomaterials for contrast enhancement, it is imperative to also consider the material chemical composition, projected human dose, interactions with biological tissue, clearance and most importantly the safety of these agents.^107^ These factors must also be aligned carefully with the imaging system, to fully appreciate detection limits.^108^

Lessons from the development of magnetic resonance imaging strongly indicate that equipment should evolve simultaneously with the chemical and biological development of the nanomaterial. Currently the only safe nanomaterial for contrast enhancement in clinical development is the iron oxide nanoparticle. Ferumoxytol (Feraheme®) (a 30 nm carbohydrate-coated superparamagnetic iron oxide NP) that has been approved by the FDA in 2009 for iron replacement.^109,110^ Given this regulatory approval and the potential that ferumoxytol has demonstrated in MRI, it seems likely that iron oxide NPs will be tested in clinical trials of MW imaging and hyperthermia.

While well-characterised for MW absorption properties, questions remain over the biocompatibility of carbon nanotubes and therefore their ability to be successfully used as contrast agents. Despite this, it is difficult to envisage that carbon nanotubes will be tested in a clinical setting as their toxicological profile indicates that the risks outweigh the benefit.^111^ Should suitable modification for biocompatibility and clearance be achievable however, it is clear from their unique properties that these nanomaterials should be investigated for MW contrast enhancement and hyperthermia. As a further challenge to development, the various biocompatible versions of CNTs would have to be continuously monitored throughout to ensure that the chemical functionalisation does not affect their properties of absorbing MW energy.

Devices based on MW technologies can provide treatment through modification of the tissue temperature. In oncology, hyperthermia is used to sensitize tumour cells, making the cancerous tissue more susceptible to chemotherapy and radiotherapy. The key challenge with MW hyperthermia devices is the inability to focus as they apply hyperthermia regionally within tissues, and this continues to be a disadvantage for devices currently in development. Nanomaterials that absorb MW radiation could be used to overcome this hurdle through their selective accumulation in the target area, which would provide a temperature increase in this region. The result being a focused and targeted response to applied MW radiation.

For MW to achieve hyperthermic or ablative temperatures, iron oxide NPs could be used as they have previously shown promise.^84^ Interestingly, the temperature increase of the tumour can be controlled even further using certain types and sizes of iron oxides. The application of iron oxide NPs with MW hyperthermia is one that has attracted significant attention, mainly due to the known safety of iron oxide NPs. Various methods are suggested for the design of NP assisted MW hyperthermia including computational methods.^112^

In a similar way, gold nanoparticle-enhanced MW hyperthermia could offer better clinical translation due to the biocompatibility of the gold and its potential to induce more local effects such as the production of free radicals.^94,113^

Overall, there are a number of materials that have shown promise in the development of nanoparticle-enhanced MW imaging and hyperthermia. There are, however, challenges that need to be overcome, primarily a firm knowledge base in the relative permittivity of both healthy and malignant tissues. Current data are limited and based on excised tissues from surgeries. The accuracy of these measurements has been called into question due to the fact that tissue hydration plays an important role in its dielectric constant.^114^ The second challenge that scientists need to address is the direct correlation between the tumour NP concentration, NP and tissue relative permittivity and MW frequencies.

Recent improvements in MW devices have allowed progression to clinical trial in applications such as breast cancer. Sensitivity of MW imaging devices could be increased with companion NPs, with candidates such as iron oxides and ferrites, to alter the dielectric properties. Similar NPs could be used for MW hyperthermia, controlling this way the increase of the temperature only in the targeted lesion. As with any emerging technology, more research is required to support success and address unmet medical need. Running parallel with this is the ongoing development in MW hyperthermia, with associated microwave-sensitive materials that seek to improve therapeutics, particularly in combination with controlled drug release. As the field moves in this direction, the potential for combined imaging and targeted therapy through the use of NPs has become the goal, offering a new clinical outlook for MW research.

## Conflicts of interest

There are no conflicts to declare.

## Supplementary Material
